# Efficacy and safety of pharmacological agents in the treatment of erythema migrans in early Lyme borreliosis—systematic review protocol

**DOI:** 10.1186/s13643-016-0251-3

**Published:** 2016-05-03

**Authors:** Gabriel Torbahn, Heidelore Hofmann, Roman Allert, Michael H. Freitag, Rick Dersch, Volker Fingerle, Harriet Sommer, Edith Motschall, Jörg J. Meerpohl, Christine Schmucker

**Affiliations:** Cochrane Germany, Medical Center—University of Freiburg, Berliner Allee 29, Freiburg, 79110 Germany; Department of Dermatology and Allergology, Technical University of Munich, Munich, Germany; Department of Obstetrics and Gynecology, University of Frankfurt, Frankfurt, Germany; Department of Health Services Research, University of Oldenburg, Oldenburg, Germany; Department of Neurology, Medical Center—University of Freiburg, Freiburg, Germany; German National Reference Centre for Borrelia—Bavarian Health and Food Safety Authority (LGL), Oberschleißheim, Germany; Institute for Medical Biometry and Statistics, Medical Center—University of Freiburg, Freiburg, Germany

**Keywords:** Erythema migrans, Lyme borreliosis, Skin manifestation, Pharmacological treatment, RCT, Non-RCT, Systematic review, Decision-making

## Abstract

**Background:**

Erythema migrans represents an early cutaneous and most common manifestation of Lyme borreliosis. Recommendations regarding pharmacological agents, dose and duration of treatment are subject of intense debate. This review aims to explore differences in efficacy and safety between pharmacological treatments and control treatment.

**Methods:**

To identify relevant studies, we will conduct a systematic literature search. We will include randomised controlled trials (RCTs) and non-RCTs. Eligible comparative studies need to (1) consider patients with a diagnosis of erythema migrans resulting from Lyme borreliosis and (2) compare different pharmacological agents against each other, against any other non-pharmacological treatment, placebo or no treatment. Two review authors will independently assess included studies for risk of bias according to the methods of the Cochrane Handbook for Systematic Reviews of Interventions and related to specific study designs. We will address patient-relevant outcomes including clinical remission of cutaneous symptoms, any treatment-related adverse events, quality of life and progressive symptoms such as neuroborreliosis or Lyme carditis and flu-like symptoms. Provided that the identified trials are comparable in terms of clinical issues, combined estimates will be provided. Estimations of treatment effects will be calculated based on a random effects model. Heterogeneity will be evaluated based on *I*^*2*^ and chi-square test. In case of significant heterogeneity, a pooled estimate will not be provided, but heterogeneity will be investigated on the basis of methodological and clinical study aspects. We plan subgroup analysis to reveal potential differences in the effect estimates between patient populations and treatment specifications. We will consider risk of bias using sensitivity analyses to decide whether to rely on the pooled estimates. The quality of a body of evidence for individual outcomes will be assessed using the GRADE approach.

**Discussion:**

Benefits and harms of pharmacological treatment in erythema migrans have not yet been adequately assessed. This systematic review will evaluate and summarise available evidence addressing benefits and harms of different pharmacological treatments. In addition, this summary of clinical evidence will inform decision-making between clinicians and patients and will play an important part in patient care.

**Systematic review registration:**

PROSPERO: CRD42016037932

**Electronic supplementary material:**

The online version of this article (doi:10.1186/s13643-016-0251-3) contains supplementary material, which is available to authorized users.

## Background

Lyme borreliosis (Lyme disease) is an infectious disease caused by spirochetes and transmitted by tick bites. *Borrelia burgdorferi s. l. complex* infection is known in most northern hemisphere countries in Europe, USA and Asia according to the distribution of the respective Ixodes ticks [1–6]. Clinical manifestations of Lyme borreliosis are split into early localised (such as erythema migrans (EM, also known as erythema chronicum migrans), borrelial lymphocytoma), early disseminated (multiple erythemata migrantia (MEM), early neuroborreliosis, Lyme carditis) and late manifestations (such as acrodermatitis chronica atrophicans, late neuroborreliosis, Lyme arthritis). From the primary skin manifestation, *Borrelia burgdorferi* can disseminate to other organs.

The typical clinical sign of early skin infection with *B. burgdorferi* is a circular, outwardly expanding red patch with central clearing called EM. Typically EM occurs at the site of the tick bite after three to 32 days [7–10]. EM associated with early infection is found in about 70–90 % of patients [5, 11, 12] and can have a range of appearances including the typical bull-s-eye lesion, but atypical lesions are also common [7–9, 13, 14]. People affected with Lyme borreliosis can also experience flu-like symptoms, such as headache, muscle and joint paints, fever and malaise [15]. The current literature provides no standardised criteria for the diagnosis of Lyme borreliosis but case definitions for surveillance by the Centers for Disease Control and Prevention (CDC) [16] or epidemiological issues by the European concerted action on Lyme borreliosis (EUCALB) [10] were provided.

Particularly, untreated early localised infection with *B. burgdorferi* can progress to disseminated manifestations such as neuroborreliosis in 10–20 % of patients [3, 5]. Other disseminated manifestations of Lyme borreliosis include Lyme carditis, present in <1 % of patients in Europe [3, 17] and <10 % of patients in the USA [18], Lyme arthritis, present in 2–7 % of patients in Europe [3] and <10 % in the USA [17] or acrodermatitis chronica atrophicans, occurring in <5 % of patients in Europe [3, 17, 18].

Antibiotic treatment represents the first-line therapy in Lyme borreliosis. Thereby, the most common agents in use are doxycycline, amoxicillin, cefuroximaxetil, azithromycin and penicillin. While probenecid was used as co-administration in treatment with amoxicillin to increase bioavailability in trials conducted in the USA, probenecid is not used anymore when treating with amoxicillin.

While the duration of treatment of persistent symptoms attributed to borreliosis is major area of debate, there is an overall consensus in favour of antibiotic treatment but dosage and duration of treatment is still subject of debate. For example, the guidelines of the Infectious Disease Society of America (IDSA) [18] and the German Dermatological Society (DDG (Deutsche Dermatologische Gesellschaft)) [19] recommend doxycycline 100 mg twice a day for 14 to 21 days, whereas the International Lyme and Associated Diseases Society (ILADS) recommends a more extended treatment duration of doxycycline for up to 42 days [20]. The ILADS also emphasises that antibiotic treatment regimens of 20 or fewer days should not be given to patients with EM because failure rates may be unacceptably high. In contrast to the most common prescribed dose of 200 mg per day, the German Borreliosis Society (Deutsche Borreliose Gesellschaft (DBG)) recommends a higher dosage of 400 mg [21]. Particularly in Europe, amoxicillin is also commonly used. Different clinical trials demonstrated that it is effective if given in a dose of 500 mg three times a day in patients with EM [22, 23]. However, treatment recommendations for amoxicillin also differ between different guidelines. For example, the ILADS recommends a total dose of 1500 to 2000 mg a day for 28 to 42 days, while the DBG recommends a much higher dose of up to 6000 mg a day for at least 28 days. In contrast, the guidelines of the DDG and IDSA recommend doses between 500 and 1000 mg three times a day for only 14 to 21 days. The use of combinations of antibiotics is advocated by the guideline of the DBG [21] whereas it is not recommended by any of the others (such as the IDSA guideline [18]).

It is obvious that conflicting guidelines often arise when evidence is weak; different methods for guideline development are used by the organisations or societies or when benefits and harms of interventions are valued differently by guideline developers [24]. In regard to the above presented controversies, a concise risk-benefit analysis in form of a rigorous conducted systematic review could greatly influence decision-making by clinicians to offer the most effective treatment to this patient population. Due to the fact that Lyme borreliosis may be associated with different manifestations (see above) for which a wide range of pharmacological treatment options is available, we plan a series of reviews on Lyme borreliosis. Shortly, a review on neuroborreliosis has already been published by our group [25, 26]. This systematic review will, therefore, be the second of a series of reviews addressing efficacy and safety of pharmacological treatments of adults in different clinical manifestations of Lyme borreliosis.

## Methods

### Objectives

This review aims to explore differences in efficacy and safety between pharmacological treatments and compared to control treatment in adults with EM. We conducted this protocol according to the PRISMA-P guideline [27]. The checklist can be found in the Additional file 1.

### Types of studies

We will include randomised controlled trials (RCTs) and non-RCTs. For non-RCTs, we will consider both cohort and case-control studies. Eligible comparative studies need to (1) consider patients with a diagnosis of EM and (2) compare different pharmacological agents against each other, against any other non-pharmacological treatment, placebo or no treatment. We will also include case series, but they have to report a minimum of 10 patients and report safety data (or—in our case—progressive patient-relevant symptoms related to the disease).

We are interested in data from non-RCTs and case series, because it is assumed that safety data (e.g. rare adverse effects or progressive symptoms) from these study types might show a greater external validity than data from RCTs [28]. In RCTs, data on adverse effects might be underestimated, as small sample sizes (<1000) limit the ability to detect rare but serious events. In addition, RCTs often include highly selected patients in which rare adverse effects, which may be associated with patients’ comorbidities or pharmacovigilance, are rarely detected. Therefore, in RCTs large sample sizes are needed to identify differences between the treatment groups and/or establish causality between an adverse outcome and the intervention [29]. In contrast, non-RCTs are often based on large databases and long follow-up times. Therefore, it is more likely that rare adverse effects or progressive symptoms related to Lyme borreliosis for a wide range of patients might be detected with these study types [29].

We will not apply any other restrictions.

### Types of participants

We will include studies where adults (at least 80 % of study population ≥18 years of age) with a clinically, (physician-confirmed) early localised skin infection (EM) were treated with pharmacological agents (at least in the intervention group). As there are no objective standard diagnostic criteria for EM available, we will rely on the definitions for EM provided in the clinical studies. Patients diagnosed with late or disseminated manifestations of Lyme borreliosis (such as disseminated EM or neuroborreliosis) will be excluded from this review.

### Types of interventions

We will evaluate any pharmacological agents (such as antibiotics, steroids, analgesics and phytotherapeutic agents), applied in any dose and in any treatment interval.

Comparative studies need to compare different pharmacological agents against each other, against any other non-pharmacological treatment, placebo or no treatment.

### Types of outcome measures

We will address the following patient-relevant outcomes:

- Remission of cutaneous symptoms (healing rate, improvement): We acknowledge that eradication of the bacterium would be the most preferable outcome in the treatment of EM. However, the proof of Borrelia eradication by direct detection (culture, PCR) or by detection of *B. burgdorferi*-specific antibodies is challenging and not sufficiently reliable. Therefore, we consider ‘clinical improvement of EM’ as the most important patient-relevant outcome for our research question. If remission is defined other than healing rate or improvement of cutaneous symptoms in the included studies, we will use the change of symptoms described by the authors of the original studies.
- Any treatment-related adverse events: We will distinguish between minor adverse events (such as diarrhea, nausea or Herxheimer reaction) and serious adverse events (such as mortality, morbidity or hospitalisation); we will use the adverse events definitions described by the authors of the original studies.
- Patient-reported outcomes: The main patient-reported outcome of interest is health-related quality of life (such as reported by validated scales (SF-36 [30])). Other relevant patient-reported outcomes that will be considered are fatigue, pain, depression, cognition and sleep.
- Progressive symptoms after treatment: Treatment failure may result in different manifestations of Lyme borreliosis (such as neuroborreliosis, Lyme carditis, Lyme arthritis, acrodermatitis chronica atrophicans, and flu-like symptoms [10, 18]); reported symptoms should be measured by validated scales (such as via the Neurological Impairment Scale (NSI) [31]; disability should be measured with the modified ranking scale [32]). When information is lacking or no valid method was used, which is assumed to be the case in the majority of studies, outcomes will be considered as defined by the authors of the original publication. Lack of validated measurement of outcomes will be considered in the risk of bias assessment and robustness of data evaluated in sensitivity analyses.

If several time points are reported in a primary study, data from the last reported time point will be considered. If data permits, results will be presented for short-term follow-up (6 weeks following start of treatment) and for long-term follow-up (more than 6 weeks following the start of treatment).

### Search methods for identification of studies

To identify relevant studies we will conduct electronic searches in Medline (via Ovid, from 1950 to present), Medline in process and other non-indexed citations (via Ovid), Medline daily update (via Ovid), Embase (via Ovid, from 1980 to present) and the Cochrane Central Register of Controlled Trials. Additionally, we will check reference lists of included studies and contact experts.

Search for ongoing trials or trials completed but not published will be conducted in ClinicalTrials.gov (www.clinicaltrials.gov) and the WHO International Clinical Trials Registry Platform (ICTRP) (http://www.who.int/ictrp/search/en).

The search strategies for the databases mentioned are shown in Additional file 2. No language restrictions will be set.

### Data collection and analysis

#### Selection of studies

Firstly, one reviewer will evaluate the titles and abstracts to determine whether a study possibly meets the eligibility criteria. Secondly, full texts of all possibly eligible studies will be retrieved and evaluated independently by two reviewers for eligibility. Disagreements will be resolved by discussion with a third reviewer. For screening, records will be imported into the reference managing software Endnote X7 (Thomson Reuters).

#### Data extraction and management

Two review authors will independently extract data from the full texts of included studies using a specifically developed data extraction form. The data extraction form will be piloted.

Information will be collected on the following items:

- Study characteristics (first author, geographical origin of study setting, year of publication, start and end of study, study design, number of arms, sample size, duration of follow-up)
- Participant characteristics (age, sex, numbers of participants, place of residence, establishment of diagnosis, diagnostic results, case definitions, disease manifestations, inclusion and exclusion criteria in the included studies, baseline imbalances between study arms and possible confounders (number of patients with disseminated infections at study entry, delay between onset of symptoms and treatment, previous treatment, baseline level of antibodies, co-medication, co-morbidities and other confounders as reported by the authors))
- Intervention and comparator details (sample size for each treatment arm, dose and type of interventions, route of delivery, dose adjustment based on body weight, duration of treatment, withdrawals and drop-outs)
- Outcome measures (description of assessment tools used, data for continuous/dichotomous/categorical efficacy variables, unit of measurement, upper and lower scale limits, collected and reported time points of measurement).

When adjusted analyses are available in primary studies, these adjusted estimates of treatment effects will be used. Otherwise, we will extract the unadjusted data as reported in the primary study. Data will be entered into Review Manager (RevMan 5.3) by one of the reviewers and checked by a second reviewer [33].

Discrepancies in data extraction or entry will be resolved by discussion with a third reviewer. Reviewers will not be blinded to study author, journal or institution.

#### Assessment of risk of bias

The assessment of risk of bias (RoB) will be performed by two reviewers independently considering the following domains according to the Cochrane risk of bias tool for RCTs: sequence generation, allocation concealment, blinding (of participants, personnel and outcome assessors), incomplete outcome data, selective outcome reporting and other sources of bias for the RCTs [34]. According to the Cochrane Handbook, these items will be described as having ‘low’, ‘high’ or ‘unclear’ RoB [34]. Bias in comparative studies will be evaluated separately. The following domains will be addressed for non-RCTs: bias due to confounding (such as age, time from onset of symptoms until treatment), bias in selection of participants into the study, bias in measurement of interventions, bias due to departures from intended interventions, bias due to missing data, bias in measurement of outcomes, bias in selection of the reported result and overall bias. These will be assessed according to the ‘Cochrane risk of bias tool for non-randomised studies’ (ACROBAT-NRSI) [35]. According to the ACROBAT-NRSI, these items will be judged as ‘low’, ‘moderate’, ‘serious’, ‘critical’ or ‘unclear’ risk of bias [35]. At the time of writing this protocol, this tool is being revised.

We expect that the major confounders which could influence effect measures in EM are ‘time from onset of symptoms until treatment’, ‘co-morbidities’, ‘intake of drugs that are not part of treatment’, ‘geographical origin’ and ‘age’. ‘Time from onset of symptoms until treatment’ is included in the ACROBAT-NRSI tool in the domain ‘bias due to confounding’. In the event of disagreement, consensus will be achieved through a discussion with a third reviewer. According to the recommendations for the ACROBAT-NRSI, no studies assessed as having a ‘critical’ RoB will be included in any data synthesis.

#### Measures of treatment effect

We will analyse outcomes measured with a scale as continuous outcomes. If more than one scale is used to measure an outcome in the very same study, only the measurement on a validated scale will be considered. In the case when more than one validated or more than one but only scales that are not validated are reported for one outcome, we will use the results provided by the scale which is mostly used in the other included studies.

The treatment effect for each continuous outcome (such as improvement of EM, patient-reported outcomes such as quality of life, progressive symptoms such as headache or complications such as disability) will be expressed as a mean difference (MD) with 95 % confidence interval (CI). Where continuous outcomes are measured using different scales, the treatment effect will be expressed as a standardised mean difference (SMD) with 95 % CI. As recommended by Guyatt et al., when possible, the treatment effects will be additionally expressed by the ratio of means (RoM) with 95 % CI to facilitate interpretation [36, 37].

The treatment effect for dichotomous outcomes (such as presence of EM, presence of progressive symptoms or adverse events) will be expressed as a risk ratio (RR) or odds ratio (OR) in case of case-control studies with 95 % confidence intervals.

If data do not permit analysis of ‘cutaneous symptoms’ as a continuous outcome, we will use the reported data from primary studies and treat this outcome as a dichotomous outcome ‘presence of cutaneous symptoms’.

Measures of treatment effects will be pooled for RCTs and non-RCTs, separately.

#### Unit of analysis

Each patient recruited in included studies will be the unit of analysis.

#### Dealing with missing data

Data will be analysed—if possible—on intention-to-treat (ITT) basis or after recently developed approaches addressing missing data in clinical trials following the recommendations for systematic reviewers by Guyatt et al. [38–40]. We will also check trial registers or contact study authors trying to get information of missing data. If results are only reported in graphs, we will estimate the values.

#### Assessment of heterogeneity

Provided that the identified trials are comparable in terms of clinical issues (such as no major imbalances or differences in the included population or the interventions), combined estimates will be provided (depending on the study design). Thereby, estimations of treatment effects will be calculated based on a random effects model [41] and heterogeneity will be evaluated based on *I*^*2*^ and the statistical test chi square. In case there is significant heterogeneity (chi-square *p* value <0.10 or an *I*^*2*^ ≥ 75 %), a pooled estimate will not be provided [42]. But heterogeneity will be investigated on the basis of methodological (such as RoB) and clinical (such as risk profile of the study population before treatment, progressive symptoms after treatment in different control groups) characteristics.

We plan subgroup analysis to reveal potential differences in the effect estimates between patient populations and treatment specifications (such as dose, length of treatment). In addition, we will consider RoB using sensitivity analyses to decide whether to rely on the pooled estimates. The quality of a body of evidence for individual outcomes will be assessed using the GRADE approach.

#### Assessment of reporting biases

We plan to minimise the impact of reporting bias in our systematic review by ensuring a comprehensive search for eligible studies including trial registries. A funnel plot and appropriate statistical tests for small study effects will be performed if ≥10 studies are available [43].

#### Data synthesis

Data from RCTs and non-RCTs will be analysed—due to different evidence levels—separately [44]. Combined estimates (based on a random effects model [see above]) will not be provided for studies with considerable imbalances or differences in the included population or differences in interventions. Pooling of data of non-RCTs will only be considered among studies with similar design (such as prospective cohort studies will only be combined with other prospective cohort studies) and comparable patients and intervention characteristics. We will calculate pooled RRs and 95 % CIs using Review Manager (RevMan 5.3) [33]. When significant heterogeneity is found between comparable studies (chi-square *p* value <0.10 or *I*^*2*^ > 75 %, see above), pooled estimates will not be provided. Instead, a descriptive synthesis of findings will be performed.

#### Subgroup analysis and investigation of heterogeneity

We plan subgroup analyses to evaluate whether effect estimates differ between studies with different lengths of antibiotic treatment (regardless of antibiotic group) within pre-specified durations (<14 days, 14–21, >21 days). Due to the clinical importance of antibiotic treatment options, subgroup analyses focusing on different antibiotics are also of considerable interest. Therefore, we will evaluate pre-specified classes of antibiotics (such as doxycycline, amoxicillin, amoxicillin plus probenecid and cephalosporins, other antibiotics and combinations of antibiotics) in subgroup analyses. Cephalosporins and penicillins will be lumped together as beta-lactam antibiotics to be compared to other groups of antibiotics. Also, the adequate dose of antibiotic agents is of particular relevance. For example, we plan subgroup analyses for daily doxycycline doses ≤200 mg with doses >200 mg. A subgroup analysis will be performed to investigate whether treatment effects differ in relation to case definitions in the primary studies. The variety of causative organisms in Europe compared to the USA could also lead to heterogeneity; consequently, the effect of geographical origin of the study population and different causative organisms will be evaluated in a subgroup analysis.

#### Sensitivity analysis

Sensitivity analyses will be conducted to determine the impact of bias by exclusion of studies with high risk of bias. We will particularly value masking of investigators and the use of a standard classification system for outcome measures. We will also conduct sensitivity analyses excluding studies in which causes of missing data are not reported or if there is an imbalance between study arms regarding exclusions related to safety aspects or assumed treatment failures. Pooled effect estimates of studies which adjusted for confounders (low risk of bias) will be compared with pooled effect estimates of all studies (studies which adjusted and studies which did not adjust for confounders). If there are differences between these estimates, they will be considered in the results and discussion.

#### Assessing the quality of evidence

We will use the Grading of Recommendations Assessment, Development and Evaluation (GRADE) approach to assess the quality of evidence for each outcome [45]. We will judge the quality of evidence based on the suggested five criteria for down-rating our confidence in effects estimates (risk of bias, inconsistency, imprecision, indirectness, and publication bias) and the three criteria for up-rating our confidence (large effect, dose–response gradient and opposing confounding). Based on these criteria, the quality of evidence judgement can range from very low to high.

## Discussion

Controversy exists about the choice of drug, dose and length of treatment in the therapy of EM.

Evidence from RCTs on this topic are likely scarce; therefore, we will perform a comprehensive and systematic review of the evidence incorporating RCTs and non-RCTs examining pharmacological treatments for adults with EM. In this protocol, possible subgroup analyses and sensitivity analyses are predefined. Clinically important and much debated questions regarding differences in efficacy and safety of varying antibiotics and length of antibiotic treatment will be investigated. Adverse events of treatments will be evaluated, for which non-RCTs are particularly valuable.

In contrast to previous conducted reviews and guidelines including patients with EM, our systematic review will both assess RoB for all included study designs and appraise the quality of evidence for each outcome according to the GRADE approach [18, 46–48]. Our results will be important to clarify controversies and reduce uncertainty both for patients and healthcare providers. Particularly due to the inclusion of non-RCTs, which often show longer follow-ups than controlled trials, we expect good generalisability of the results of this systematic review in terms of progressive symptoms of EM and patient safety between the different treatment options. The summary and evaluation of the available body of evidence may lead to evidence-based treatment recommendations for EM.

## Additional files

Additional file 1: PRISMA-P checklist. (PDF 151 kb) Additional file 2: Search strategies. (PDF 198 kb)

## Abbreviations

ACROBAT-NRSI: a Cochrane risk of bias tool for non-randomised studies
DBG: Deutsche Borreliose Gesellschaft
DDG: Deutsche Dermatologische Gesellschaft
EM: erythema migrans
GRADE: Grading of Recommendations, Assessment, Development and Evaluation
IDSA: Infectious Disease Society of America
ILADS: International Lyme and Associated Diseases Society
RCT: randomised controlled trial
RoB: risk of bias
