# Supplementary material for: Efficacy and safety of pharmacological agents in the treatment of erythema migrans in early Lyme borreliosis—systematic review protocol
Source: Syst Rev. 2016 May 3;5:73. doi: 10.1186/s13643-016-0251-3 (PMC4855495; doi:10.1186/s13643-016-0251-3)
Supplement: Additional file 2: — Search strategies. (PDF 198 kb) [file 13643_2016_251_MOESM2_ESM.pdf]

Additional File 2: Search strategies

Database(s): Ovid MEDLINE(R), Ovid OLDMEDLINE(R)

1	exp Lyme Disease/
2	lyme*.mp.
3	exp Borrelia/
4	borreli*.mp.
5	tick*.mp.
6	(erythem* adj2 migran*).mp.
7	dermat*.mp.
8	cutan*.mp.
9	exp Erythema/
10	erythem*.mp.
11	exp Skin Diseases, Bacterial/
12	skin diseas*.mp.
13	acroderm* chron* atrophy*.mp.
14	mult* erythem* migran*.mp.
15	Scleroderma, Localized/
16	local* slcero*.mp.
17	circ* sclero*.mp.
18	morphea.mp.
19	Pseudolymphoma/
20	pseudolymphom*.mp.
21	lymphocytom*.mp.
22	(cutan* adj2 lymphocyt*).mp.
23	Lichen sclerosus/

24 Lichen sclero*.mp.

25 atroph*.mp.

26 aneto*.mp.

27 granuloma*.mp.

28 neuroborreli*.mp.

29 arthritis.mp.

30 carditis.mp.

31 early locali*.mp.

32 (early adj2 lyme*).mp.

33 (early adj2 borrel*).mp.

34 acute lyme*.mp.

35 (acute adj2 borrel*).mp.

36 early dissem*.mp.

37 late dissem*.mp.

38 late lyme*.mp.

39 (late adj2 borrel*.mp.

40 (dissem* adj2 borrel*).mp.

41 (chron* adj2 borrel*).mp.

42 chron* lyme*.mp.

43 (subacute adj2 borrel*).mp.

44 subacute lyme*.mp.

45 (refractory adj2 borrel*).mp.

46 refractory lyme*.mp.

47 or/1-5

48 or/6-12

49 or/13-30

50 or/31-46

51 48 or 50

52 49 or 50

53 47 and 51

54 47 and 52

55 53 or 54

[mp=title, abstract, original title, name of substance word, subject heading word, keyword heading word, protocol supplementary concept word, rare disease supplementary concept word, unique identifier]

Database(s): Ovid MEDLINE(R) In-Process & Other Non-Indexed Citations

1 lyme*.mp.

2 borreli*.mp.

3 tick*.mp.

4 (erythem* adj2 migran*).mp.

5 dermat*.mp.

6 cutan*.mp.

7 erythem*.mp.

8 skin diseas*.mp.

9 acroderm* chron* atrophy*.mp.

10 mult* erythem* migran*.mp.

11 local* slcero*.mp.

12 circ* sclero*.mp.

13 morphea.mp.

14 pseudolymphom*.mp.

15 lymphocytom*.mp.

16 (cutan* adj2 lymphocyt*).mp.

17 Lichen sclero*.mp.

18	atroph*.mp.
19	aneto*.mp.
20	granuloma*.mp.
21	neuroborreli*.mp.
22	arthritis.mp.
23	carditis.mp.
24	early locali*.mp.
25	(early adj2 lyme*).mp.
26	(early adj2 borrel*).mp.
27	acute lyme*.mp.
28	(acute adj2 borrel*).mp.
29	early dissem*.mp.
30	late dissem*.mp.
31	late lyme*.mp.
32	(late adj2 borrel*.mp.
33	(dissem* adj2 borrel*).mp.
34	(chron* adj2 borrel*).mp.
35	chron* lyme*.mp.
36	(subacute adj2 borrel*).mp.
37	subacute lyme*.mp.
38	(refractory adj2 borrel*).mp.
39	refractory lyme*.mp.
40	or/1-3
41	or/4-8
42	or/9-23
43	or/24-39
44	41 or 43

45	42 or 43

46	40 and 44

47	40 and 45

48	46 or 47

[mp=title, abstract, original title, name of substance word, subject heading word, keyword heading word, protocol supplementary concept word, rare disease supplementary concept word, unique identifier]

Database(s): Ovid MEDLINE(R) Daily Update

1	exp Lyme Disease/

2	lyme*.mp.

3	exp Borrelia/

4	borreli*.mp.

5	tick*.mp.

6	(erythem* adj2 migran*).mp.

7	or/1-6

[mp=title, abstract, original title, name of substance word, subject heading word, keyword heading word, protocol supplementary concept word, rare disease supplementary concept word, unique identifier]

Database(s): Pubmed MEDLINE(R) Publisher

1	lyme*[tw]

2	borreli*[tw]

3	tick*[tw]

4	erythem*[tw]

5	migran*[tw]

6	#4 AND #5

7	#1 OR #2 OR #3 OR #6

8 #7 AND publisher[sb]

[tw= Title, Abstract, MeSH, Subheadings, Publication Types, Other Terms, Chemical Names of Substances, Secondary Source Identifier, Personal Name as Subject; sb= Subset]

Database(s): Ovid Embase

1 exp Lyme Disease/

2 lyme*.mp.

3 exp Borrelia infection/

4 borreli*.mp.

5 tick*.mp.

6 (erythem* adj2 migran*).mp.

7 dermat*.mp.

8 cutan*.mp.

9 exp erythema/

10 erythem*.mp.

11 exp bacterial skin disease/

12 skin diseas*.mp.

13 acroderm* chron* atrophy*.mp.

14 mult* erythem* migran*.mp.

15 exp localized scleroderma/

16 local* slcero*.mp.

17 circ* sclero*.mp.

18 exp morphea/

19 morphea.mp.

20 exp pseudolymphoma/

21 pseudolymphom*.mp.

22 lymphocytom*.mp.

23 (cutan* adj2 lymphocyt*).mp.

24 exp lichen sclerosus et atrophicus/

25 Lichen sclero*.mp.

26 atroph*.mp.

27 aneto*.mp.

28 granuloma*.mp.

29 neuroborreli*.mp.

30 arthritis.mp.

31 carditis.mp.

32 early locali*.mp.

33 (early adj2 lyme*).mp.

34 (early adj2 borrel*).mp.

35 acute lyme*.mp.

36 (acute adj2 borrel*).mp.

37 early dissem*.mp.

38 late dissem*.mp.

39 late lyme*.mp.

40 (late adj2 borrel*.mp.

41 (dissem* adj2 borrel*).mp.

42 (chron* adj2 borrel*).mp.

43 chron* lyme*.mp.

44 (subacute adj2 borrel*).mp.

45 subacute lyme*.mp.

46 (refractory adj2 borrel*).mp.

47 refractory lyme*.mp.

48 or/1-5

49 or/6-12

50	or/13-31

51	or/32-47

52	49 or 51

53	50 or 51

54	48 and 52

55	48 and 53

56	54 or 55

[mp=title, abstract, original title, name of substance word, subject heading word, keyword heading word, protocol supplementary concept word, rare disease supplementary concept word, unique identifier]

Database(s): Central

1	MeSH descriptor: [Borrelia] explode all trees

2	MeSH descriptor: [Lyme Disease] explode all trees

3	*borreli*

4	lyme*

5	tick*

6	erythem* near/2 migran*

7	#1 OR #2 OR #3 OR #4 OR #5 OR #6

8	Trials
